# Penetrated Arrow Shot Injury in Anterior Neck

**Published:** 2011-03

**Authors:** Shuaib K. Aremu, Benjamin Dike

**Affiliations:** *Unit of Ear, Nose and Throat, Federal Medical Centre, Azare, Bauchi State, Nigeria*

**Keywords:** arrow injury, penetrating injury, anterior neck wound

## Abstract

**Background:**

Although gunshot injuries are the most common penetrating anterior neck injuries in the developed world, this finding is not the case in the developing world, where knives, spears, arrows, and machetes are the preferred weapons, particularly in tribal societies.

**Aim:**

To present the case report of a patient with arrow shot injury to the anterior neck.

**Method:**

A 48 year old cattle rearer in a village in northern part of Nigeria presented with 3 hr history of an arrow shot in anterior part of the neck which he sustained the while trying to prevent some armed robbers from stealing his cow. There was scanty bleeding from the site with the arrow in-situ sealing the wound.

**Result:**

The entrance point of the arrow, about 1 cm in diameter, was just at the anterior border of the right sternocleidomastoid muscle; about 4 cm above the medial end of the right clavicle. There was a small skin bruise with slight swelling and tenderness around the opening but no active bleeding and no crepitus.

**Conclusion:**

Penetrating neck trauma from arrow shot may lead to potentially life-threatening injuries. A prompt diagnosis, a systematic treatment protocol, and an experienced trauma team are necessary to prevent a potential catastroph.

## INTRODUCTION

Although gunshot injuries are the most common penetrating anterior neck injuries in the developed world, this finding is not the case in the developing world, where knives, spears, arrows, and machetes are the preferred weapons, particularly in tribal societies. Arrow shot found in-situ in the neck at presentation requires urgent exploration under general anesthesia. In case the arrow had been removed before patient presented, profuse bleeding from the site will also warrant urgent exploration in other to secure hemostasis.

## CASE REPORT

A 48 year old cattle rearer in a village in northern part of Nigeria presented with 3 hr history of an arrow shot in anterior part of the neck which he sustained the while trying to prevent some armed robbers from stealing his cow. There was scanty bleeding from the site with the arrow insitu sealing the wound. At presentation, there was no associated heamatemesis, cough, difficulty in breathing nor loss of speech. However, there was a small swelling around the entrance point of the arrow with moderate pain.

The physical examination revealed a middle aged man conscious not pale, with temperature of 37°C, respiratory rate of 21/min, pulse rate of 86/min and BP of 130/80 mm Hg.

**Figure 1:**
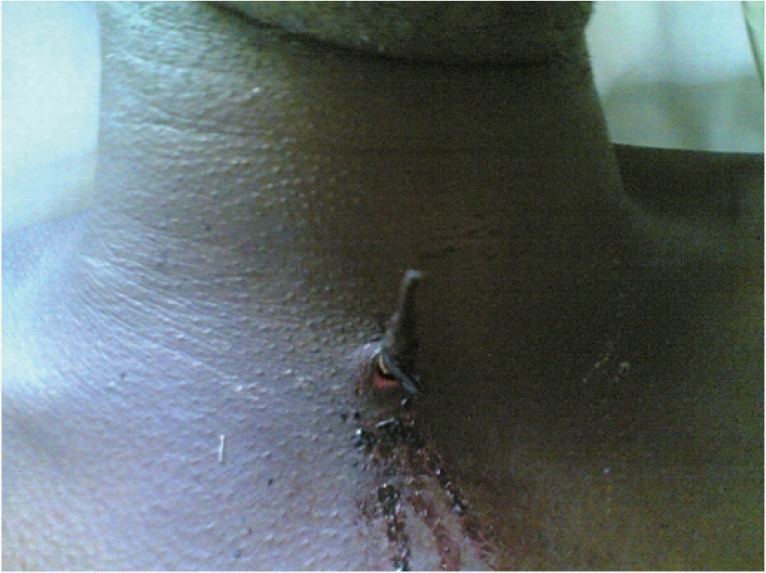
Anterior view of the neck with the arrow in-situ sealing the wound.

**Figure 2:**
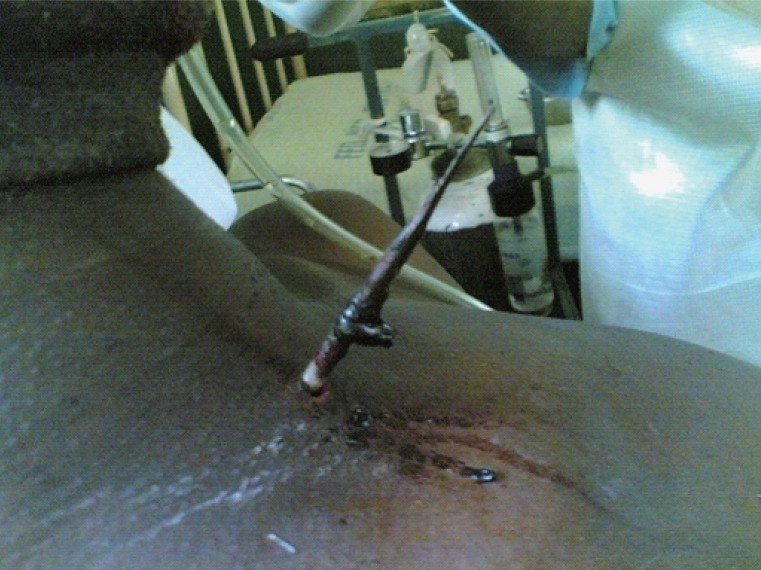
Side view of the neck with the arrow in-situ.

**Figure 3:**
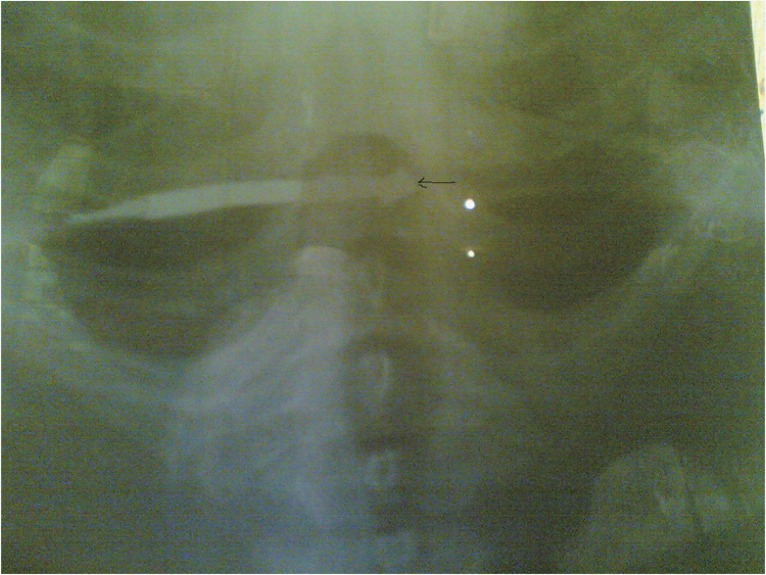
Barbed arrow tip in right tracheo-esophageal groove, pointed out by the black marker. The barbed end lying vertical.

There was an obvious distal end, 13 cm in length, of a metallic arrow in the anterior neck of the patient. The entrance point of the arrow, about 1 cm in diameter, was just at the anterior border of the right sternocleidomastoid muscle, about 4 cm above the medial end of the right clavicle. There was a small skin bruise with slight swelling and tenderness around the opening but no active bleeding and no crepitus (Fig. 1 & 2). Ear, Nose and Throat Examination were normal so were other systemic examinations. An urgent PCV done was 36%. X-rays of the neck were done which revealed the barbed arrow tip in right tracheo-esophageal groove. The barbed end lay vertical (Fig. 3).

He was commenced on intravenous fluid, cefuroxime and metronidazole. 2 units of blood was cross matched for him and he was planned for urgent anterior neck exploration and foreign body removal under general anesthesia.

At surgery, a 6 cm para-sternocleidomastoid incision was made on the skin with the entrance point of the arrow at the mid-point. The incision was deepened down and dissections done to reach the barbed end. The arrow lied in the right trachea-esophageal grove with the pointed end slightly buried in the wall of the esophagus. There was a 2 cm laceration on esophageal wall into the muscle but the mucosa was intact. Also the pre-tracheal fascia was bruised. The esophageal wall laceration was repaired in layers. No injuries to the great vessels of the neck. The wound was thoroughly irrigated with normal saline and closed in layers. A nasogastric tube (NG tube) was inserted intra-operatively.

N-G tube feeding was commenced 12 hrs after surgery for 1 week followed by peri-nasogastric tube feeding for another 5 days during which there was no evidence of mediastinitis. The tube was finally removed on the 14^th^ day. Skin suture was removed on the 5^th^ day post-op. The patient had post-operative antibiotics for 2 weeks. He was discharged on 15^th^ day post-op and had been followed up in the clinic for 2 months without complications.

## DISCUSSION

Although gunshot wounds are the most common anterior neck penetrating injuries in the developed world, this finding is not the case in the developing world, of which Nigeria is one, where knives, spears, arrows, and machetes are the preferred weapons, particularly in tribal societies. Arrow and spear wound epidemiology and management have been well described from Papua New Guinea (1, 2).

Penetrating neck injury accounts for 1% of trauma in USA (3). There is paucity of literature on arrow shot injuries in our environment thus no record of its incidence. However the incidence appears to be common among the Hausa-Fulani ethnic group of Nigeria where arrows and spears are commonly used for hunting.

Patients with arrow or spear wounds to the head and neck present in three ways: 1) The weapon is still in place. In this situation, it should be removed by a surgeon under general anesthesia so that major structures in the path of the injury can be, if possible, repaired and a catastrophe on removal avoided; 2) The weapon has been completely removed by the patient or his relatives. The underlying injuries may or may not be obvious, and usually wound exploration and debridement are necessary, particularly where CT or MRI are not available to demonstrate the extent of the damage. Late presentation with an AV fistula or false aneurysm may occur weeks after the injury; 3) Part of the weapon is retained internally, and the wound has healed. If this is not appreciated by the relatives or the primary care doctor, the opportunity for early removal is missed. The patient will return if complications develop.

Our patient presented early, after 3 hours. This unique presentation as it was unlike the typical attitude in our community where patients present late. This prevented, in this patient, serious complications of arrow injuries that have been reported in different literatures.

Experience with late complications of gunshot wounds to the head has mainly come from wars (4, 5). Late complications of penetrating arrow and spear wounds are infrequently reported but are not uncommon in Papua New Guinea where tribal fighting is still rife. The complications can be divided into vascular, infectious, and neurologic.

Radiological evaluation of the patient is important. It should be noted that not all arrow or spear tips are radiopaque. The metallic head is radiopaque as seen in our case, but CT or MRI are preferred investigations in such cases rather than plain X-ray. MRI is useful in identifying wood fragments (6, 7).

Wood carries a high risk of infection and fragmentation (8).

It is important to reiterate the advice of O’Neill et al. that arrows with barbs should be removed in an anterograde direction along the line of its trajectory to avoid snagging blood vessels and other structures (9).

Penetrating neck injuries present complex management problems for the surgical trauma team because of the major vascular, neurologic, aerodigestive tract, and soft tissue structures that are at risk for injury. There is little disagreement that patients with zone II neck injuries exhibiting signs or symptoms suggestive of significant vascular or visceral injuries must undergo mandatory neck exploration (10, 11).

The indications for mandatory neck explorations include the following: 1) continued bleeding from the wound, hematoma, or shock; 2) blood in the aerodigestive tract; 3) subcutaneous emphysema, hoarseness, or aphonia; 4) neurologic deficits; and 5) inability to observe or study the patient because of other injuries. Other authors have recommended similar protocols for mandatory neck explorations (12, 13).

The presence of a highly skilled surgical trauma team and ancillary support staff are important for a disciplined approach to manage penetrating arrow neck injuries.

## CONCLUSION

Penetrating neck trauma from arrow shot may lead to potentially life-threatening injuries. A prompt diagnosis, a systematic treatment protocol, and an experienced trauma team are necessary to prevent a potential catastroph.
